# Supplementary material for: Parametric G‐computation for compatible indirect treatment comparisons with limited individual patient data
Source: Res Synth Methods. 2022 May 16;13(6):716–44. doi: 10.1002/jrsm.1565 (PMC9790405; doi:10.1002/jrsm.1565)
Supplement: Supplementary file 1 — APPENDIX S1 Supplementary Information [file JRSM-13-716-s001.pdf]

Supplementary Material of "Parametric G-computation for Compatible Indirect Treatment Comparisons with Limited Individual Patient Data"

Antonio Remiro-Azócar[1,2], Anna Heath[1,3,4], and Gianluca Baio[1]

[1]Department of Statistical Science, University College London
[2]Quantitative Research, Statistical Outcomes Research & Analytics (SORA)
[3]Child Health Evaluative Sciences, The Hospital for Sick Children
[4]Dalla Lana School of Public Health, University of Toronto

Supplementary Appendix A: Methods compared in simulation study

Matching-adjusted indirect comparison

Matching-adjusted indirect comparison (MAIC) is implemented using the original method of moments formulation presented by Signorovitch et al.[1–4] To avoid further reductions in effective sample size and precision, only the effect modifiers are included in the weighting model. A weighted logistic regression is fitted to the AC IPD and standard errors for the A vs. C marginal treatment effect are computed by resampling via the ordinary non-parametric bootstrap with replacement,[5] with 1,000 resamples of each simulated dataset. Note that the standard version of MAIC[1–4] uses a robust sandwich estimator for variance estimation[6] that accounts for the heteroskedasticity or correlation induced by the weighting. Nevertheless, this has understated variability under small effective sample sizes in previous simulation studies,[3,7] and most software implementations of the estimator treat the weights as fixed quantities. The bootstrap approach should account for the uncertainty in estimating the weights from the data. The average marginal log-odds ratio for A vs. C is calculated as the mean across the 1,000 bootstrap resamples. Its corresponding standard error is the sample standard deviation across the resamples.

In our implementation of MAIC, we only balance the covariate means and balance these for active treatment and control arms combined. Other approaches have been proposed, such as balancing the covariates separately for active treatment and common comparator arms,[8,9] or balancing terms of higher order than means, e.g. by including squared covariates in the weight estimation to balance variances. The former approach is discouraged because it may break randomization in the IPD, distorting the balance between treatment

arms A and C on covariates that are not accounted for in the weighting, and potentially compromising the internal validity of the within-study estimate. The latter approach may increase finite-sample bias[10] and has performed poorly in recent simulation studies, in terms of both bias and precision, where covariate variances differ across studies.[8,11–13]

Given the often arbitrary factors driving selection into different trials, the mechanism for generating the simulation study data in subsection 4.2 of the main text does not specify a trial assignment model. Nevertheless, the logistic regression model for estimating the weights is the "best-case" model because it selects the "right" subset of covariates as effect modifiers. The estimated weights are adequate for bias removal because the balancing property[14,15] holds with respect to the effect modifier means. Namely, conditional on the weights, all effect modifier means are balanced between the two trials, and one can achieve unbiased estimation of treatment effects in the BC population due to conditional exchangeability over trial assignment.

In a test simulation scenario with $N = 200$, bootstrapped MAIC has a running time of approximately 2.7 seconds per simulated dataset, using an Intel Core i7-8650 CPU (1.90 GHz) processor. Computation time increases linearly with the number of bootstrap resamples.

Conventional simulated treatment comparison

The conventional version of simulated treatment comparison (STC), as described by HTA guidance and recommendations,[2] is implemented. A covariate-adjusted logistic regression is fitted to the IPD using maximum-likelihood estimation. The outcome regression is correctly specified.[a] All covariates are accounted for in the regression but only the treatment effect modifiers are centered at their mean BC values, and interaction terms are only included for the effect modifiers. The log-odds ratio estimate for A vs. C is the treatment coefficient of the centered multivariable regression, with its standard error quantifying the standard deviation of the treatment effect.

In a test simulation scenario with $N = 200$, the conventional STC has a running time of 0.02 seconds per simulated dataset.

Maximum-likelihood parametric G-computation

We consider two implementations of parametric G-computation. In the first implementation, we use maximum-likelihood estimation to fit the multivariable outcome regression. The Q-model is correctly specified. We construct the joint distribution of the four BC

[a] It is more burdensome to specify an outcome regression model than a propensity score model (for the MAIC weights). The former requires specifying both prognostic and interaction terms, whereas the latter only requires the specification of effect modifiers. In practice, one cannot typically ascertain which covariates are purely prognostic variables and which covariates are effect modifiers. Exploratory simulations show that the relative precision and accuracy of MAIC deteriorate, with respect to outcome regression, if we treat all four covariates as effect modifiers. On the other hand, the relative precision and accuracy of outcome regression deteriorate if the terms corresponding to the purely prognostic covariates are not included in the outcome model. Nevertheless, the other terms in the regression already account for a considerable portion of the variability of the outcome, and relative effects are accurately estimated in any case. These alternative setups do not alter the conclusions of the simulation study.

covariates by simulating these from a multivariate Gaussian copula. This uses normally-distributed marginals with the BC means and standard deviations, and the pairwise linear correlations of the AC IPD. $N^* = 1000$ subject profiles are simulated for the BC pseudo-population, a value high enough to minimize sampling variability and provide an adequate degree of precision. Outcomes in the BC population are predicted by plugging the simulated covariates into the maximum-likelihood fit. The procedure is resampled using the ordinary non-parametric bootstrap with replacement, with 1,000 resamples of each simulated dataset. Increasing further the number of resamples produces minimal gains in estimation precision and accuracy, with the Monte Carlo error across different random seeds remaining relatively insensitive to these increases. The average marginal log-odds ratio for A vs. C is calculated as the mean across the 1,000 bootstrap resamples. Its corresponding standard error is the sample standard deviation across the resamples.

In a test simulation scenario with $N = 200$, parametric G-computation using maximum-likelihood estimation has a running time of approximately 3.5 seconds per replicate. Computation time increases linearly with the number of bootstrap resamples.

Bayesian parametric G-computation

In the second implementation of parametric G-computation, we use MCMC simulation to fit the outcome regression. This is implemented using the package `rstanarm`,[16] a high-level appendage to the `rstan` package,[17] the `R` interface for `Stan`.[18] Again, the Q-model is correctly specified. The joint distribution of the BC covariates is constructed by simulating $N^* = 1000$ subjects from a multivariate Gaussian copula, with normally-distributed marginals with the BC means and standard deviations, and the pairwise linear correlations of the AC IPD. Predicted outcomes for the simulated covariates are drawn from their posterior predictive distribution.

We use the default independent "weakly informative" priors for the logistic regression intercept and predictor coefficients, i.e., the likelihood dominates under a reasonably large amount of data and the prior strongly influences the posterior if the data are weak.[19] These are normally-distributed priors centered at mean 0. The scale of the normal prior distribution for the intercept is 1. The scale parameter of the normal priors for the other coefficients is 2.5, rescaled in terms of the standard deviation of the predictor in question. This places most of the prior mass in the range of plausible effects, discarding coefficient values that are implausibly strong, e.g. log-odds ratios over 3 (corresponding, approximately, to odds ratios over 20). This provides some regularization and helps stabilize computation. Alternative prior specifications are considered to check that we are not incorporating any unintended information into the models through the priors. Results are robust to the definitions of the prior distributions.

We run two Markov chains with 4,000 total iterations per chain. These include 2,000 warmup/burn-in iterations for each chain that are not used for posterior inference. This gives a total of 4,000 iterations for performing the analysis. Approximate mixing of the chains was attained, with all within-chain relative to between-chain statistics (R-hat) below 1.1.[20] Satisfactory convergence was confirmed by the inspection of trace plots and the assessment of diagnostics such as the effective sample size and the Gelman-Rubin convergence diagnostic (potential scale reduction factor).[20] The average marginal treatment

effect for A vs. C is estimated taking the sample mean of the marginal log-odds ratio across the 4,000 MCMC iterations. The corresponding standard error is estimated using the sample standard deviation of the posterior draws of the marginal log-odds ratio.

In a test simulation scenario with $N = 200$, Bayesian parametric G-computation has a running time of approximately 4.2 seconds per replicate. Computation time increases linearly with the total number of MCMC iterations.

Indirect treatment comparison

For all methods, the marginal log-odds ratio for B vs. C is estimated directly from the event counts, and its standard error is computed using the delta method.[21] The marginal log-odds ratio estimate for A vs. B and its standard error are obtained by combining the within-study point estimates, as per subsection 3.6 of the main text (using Equation 1 of the main text to compare point estimates and Equation 14 of the main text to sum the point estimates of the variance). Wald-type 95% interval estimates are constructed for the marginal A vs. B treatment effect using normal distributions.

In Bayesian G-computation, we have used a two-step approach for: (1) the population-adjusted analysis of the AC trial (estimation of the marginal effect for A vs. C); and (2) the indirect treatment comparison (estimation of the marginal effect for A vs. B). We also consider integrating the two in one stage, using MCMC sampling. In this case, for estimation of the marginal log-odds ratio for B vs. C, the true underlying event rates/proportions for the treatments are given non-informative Jeffreys Beta$(0.5, 0.5)$ priors. The number of events in each arm is sampled from two independent Binomial likelihoods, parametrized by the aforementioned event probabilities and the total number of subjects in each arm. Means and variances for the marginal A vs. B treatment effect are obtained empirically from the posterior samples, with interval estimates calculated from the quantiles of the posterior distribution.

While a Bayesian inferential framework might be convenient in the context of probabilistic sensitivity analysis, the selected inferential framework has little bearing on computation time and on the results of the simulation study, both in terms of a single case study and of the long-run frequentist statistical properties of parametric G-computation. Integrating the indirect treatment comparison step within a Bayesian module leads to virtually identical performance measures than the two-step approach. Therefore, results are not reported.

Supplementary Appendix B: Extended discussion

Method assumptions

Population-adjusted indirect comparisons mostly depend on the same assumptions, including: (i) internal validity of the AC and BC trials, (ii) consistency under parallel studies, (iii) accounting for all effect modifiers of treatment A vs. C in the adjustment (i.e., the conditional constancy of the A vs. C marginal treatment effect or the conditional ignorability, unconfoundedness or exchangeability of trial assignment/selection for such treatment effect), (iv) that there is overlap between the covariate distributions in AC and

BC (more specifically, that the ranges of the selected covariates in the AC trial cover their respective moments in the BC population), (v) that the joint covariate distribution of the BC population has been correctly specified, (vi) and parametric modeling assumptions.

Assumptions (i) and (ii) are made by any indirect treatment comparison or meta-analysis. The other, largely untestable, assumptions are unique to population-adjusted analyses and their violation may lead to bias. The most crucial assumptions underlying population-adjusted indirect comparisons relate to the correct specification of the trial assignment logistic regression (in the case of MAIC), and of the covariate-adjusted outcome regression (in the case of conventional STC and parametric G-computation).

In practice, there will be model misspecification if there is incomplete information on effect modifiers for one or both of the trials. Conditional exchangeability ("no omitted effect modifiers") is a fundamental assumption for all methods. However, it is not directly testable with the available data due to the lack of additional individual-level outcome information for the BC study.[22] In collaboration with clinical experts, the most plausible effect modifiers should be selected for the base-case analysis. Nevertheless, the effect modifier status of covariates is difficult to ascertain, particularly for novel treatments with limited prior empirical evidence and clinical domain knowledge.[23] Therefore, we will never be completely certain that all effect modifiers have been accounted for, or of the validity of the population adjustment.

Consequently, sensitivity analyses are warranted under alternative model specifications to explore the dependence of inferences on the model and the robustness of results.[24,25] In the context of "generalizability", Nguyen et al.[24] have recently developed an approach for sensitivity analysis. This is applicable where potential effect modifiers are measured only in the AC trial but not in the BC study, given some assumptions about the missing effect modifiers. Dahabreh[26] proposes a bias function strategy for sensitivity analyses, which does not require individual-level information on unobserved effect modifiers. Further research should adapt this recent work to our "limited patient-level data" setup.

Parametric modeling assumptions will not hold under incorrect model specification, e.g. in the outcome regression methods, if only linear relationships are considered and the selected covariates have non-linear interactions with treatment on the linear predictor scale. The simulation study only considers a best-case scenario with correct parametric model specification. To predict the outcomes, we use the logistic regression model implied by the data-generating mechanism. Similarly, the model for estimating the weights is the best-case model in MAIC because the right subset of covariates has been selected as effect modifiers and the balancing property holds for the weights with respect to the effect modifier means, as mentioned in Appendix A of the Supplementary Material. Also, effect modification has been correctly specified as linear, but scale conflicts would arise if effect modification status, which is scale-specific, had been justified on the wrong scale, e.g. if the true treatment effect modification were non-linear or multiplicative, e.g. age in cardiovascular disease treatments.

In real applications, these modeling assumptions are difficult to hold because, unlike in simulations, the correct specification is unknown, particularly where there are a large number of covariates and complex relationships exist between them. The simulation study presented in this article demonstrates proof-of-concept for the outcome regression meth-

ods and for MAIC, but does not investigate how robust the methods are to failures in assumptions. Future simulation studies should explore performance in scenarios where assumptions are violated, in order to draw more accurate conclusions with respect to practical applications and limitations.

The general-purpose nature of the G-computation methods presented in this article may provide some degree of robustness against model misspecification because the covariate-adjusted outcome model does not necessarily need to be parametric. We have considered the nuisance model in Equation 3 of the main text to be a parametric regression. Alternatively, non-parametric regression techniques or other data-adaptive estimation approaches can be used to detect (higher-order) interactions, product terms and non-linear relationships, offering more flexible functions to predict the conditional outcome expectations. These may enhance the likelihood of correct model specification with respect to parametric regressions, but are susceptible to overfitting, particularly with small sample sizes. They can also minimize "data snooping" problems (e.g. the analyst selecting the model specification or the effect modifiers on the basis of statistically significant treatment effects), especially when there are no clear hypotheses about effect modification ex ante.

In the main text, we have postulated a single outcome model for all subjects in the AC IPD, which includes the necessary treatment-covariate interaction terms to capture effect modification over the covariates. Nevertheless, another possible strategy is to fit two outcome models separately for each treatment group in the randomized trial, i.e., to fit one regression to the patients under treatment A and then another regression among the patients under C, then predicting the conditional outcome expectations and averaging these out on the entire simulated pseudo-population. In this approach, the model-fitting is performed independently of reference to a conditional treatment effect (the fitted regressions do not have a treatment coefficient), and the estimation of treatment-by-covariate interactions is obviated.[27]

Specification of the BC population Ideally, the BC population should be characterized by the full joint distribution of covariates. However, the restriction of limited IPD makes it unlikely that the joint distribution of the BC covariates is available. Where there are not many covariates and these are binary, this is sometimes available as a cross-tabulation. However, most of the time we need to approximate the joint distribution appropriately. This is important to avoid bias arising from the incomplete specification of the BC population.

Population-adjusted indirect comparisons make certain assumptions to approximate the joint distribution of covariates in the BC trial, but these assumptions differ slightly. In MAIC, as stated in the NICE Decision Support Unit technical support document,[2] "when covariate correlations are not available from the (BC) population, and therefore cannot be balanced by inclusion in the weighting model, they are assumed to be equal to the correlations amongst covariates in the pseudo-population formed by weighting the (AC) population." In the conventional version of STC, the correlations between the BC covariates are assumed to be equal to the correlations between covariates in the AC trial.

In the marginalization methods proposed in this article, more explicit and stringent distributional assumptions are made in the "covariate simulation" step. The published

summary values $\boldsymbol{\theta}$ and the correlation structure $\boldsymbol{\rho}$ are combined, making certain parametric assumptions about the marginal distributional forms, to infer the joint distribution of the BC covariates and construct an appropriate pseudo-population for inferences. The methods assume the joint distribution of the BC covariates is specified correctly, by the combination of the specified marginal distributions and correlation structure. In the simulation study, we have assumed that the pairwise correlations of the covariates and the parametric forms of their marginal distributions are identical across trials. It is important to assess the robustness of the methods to failures in these distributional assumptions.

Note that the covariate distributional assumptions could be relaxed or verified empirically if trial publications included more complete summary statistics, e.g. information on the covariates' correlation structure or their observed marginal distributions, as opposed to simple summary tables of means/proportions and standard deviations. This information would allow us to approximate the full joint distribution of the BC covariates more accurately and reduce the risk of misspecifying the BC population. We have decided to mimic the AC pairwise correlations as, in principle, the relationships between covariates should be similar across trials.

Bayesian modularity The G-computation methods, particularly the Bayesian formulation, can be readily adapted to address missing values in the AC IPD. Bayesian G-computation follows very closely the principles of multiple imputation, which is also, arguably, a fundamentally Bayesian operation. Missing covariates and outcomes in the IPD could be imputed in each MCMC iteration, accounting naturally for the uncertainty in the missing data. Addressing "missingness" in the BC study is not possible task without access to the patient-level data.

Throughout the text, we have made certain assumptions about the covariate distribution in the BC population. We have treated the covariate moments $\boldsymbol{\theta}$ and the correlation information $\boldsymbol{\rho}$ as fixed. The Bayesian framework could be extended to account for this additional layer of uncertainty, in the specification of $\boldsymbol{\theta}$ and $\boldsymbol{\rho}$ and also in the selected marginal distribution forms for BC. Bayesian regression approaches can also account for other issues such as measurement error in the IPD.[28] Bayesian model averaging can be incorporated to capture structural or model uncertainty.[29] By drawing outcome predictions under various models, complex relationships in the patient-level data may be reproduced more accurately, offering some protection against parametric model misspecification.

In the Bayesian approach, both "hard" (e.g. the results of a meta-analysis) and "soft" (e.g. clinical rationale from experts) evidence can be used to form the prior distributions for the conditional prognostic and interaction effects. The specification of the parametric outcome model requires "dichotomizing" whether a variable is an effect modifier or not, i.e., in statistical terms, specifying whether interactions with treatment do or do not exist. Bayesian shrinkage methods allow interactions to be "half in, half out" of the model.[30–32] For instance, one can specify skeptical or regularization prior distributions for the interaction effects, over all potential candidate effect modifiers. In the words of Simon and Freedman,[32] this "encourages the quantification of prior belief about the size of interactions that may exist. Rather than forcing the investigator to adopt one of two extreme positions regarding interactions, it provides for the specification of intermediate

positions".

Supplementary Appendix C: Cox proportional hazards regression

The most popular outcome types in applications of population-adjusted indirect comparisons are survival or time-to-event outcomes (e.g. overall or progression-free survival), and the most prevalent measure of effect is the (log) hazard ratio.[33] Therefore, developing G-computation approaches where the nuisance model is a Cox proportional hazards regression is important and useful to practitioners. In this setting, $\hat{\Delta}_{10}^{(2)}$ and $\hat{\Delta}_{20}^{(2)}$ should target marginal log hazard ratios for indirect treatment comparisons in the linear predictor scale. Something to bear in mind is that, even if Cox models are very frequently used in evidence synthesis for time-to-event data, health economic modelers typically use parametric survival models for extrapolation purposes.

The G-computation formulae for the Cox regression are provided by Stitelman et al.[34] Consider that a Cox proportional hazards model has first been fitted, conditional on covariates which follow the functional form in the linear predictor of Equation 3 in the main text. For the generalized linear model, we were interested in the average outcome predictions in the natural scale. With Cox regression, the average survival probabilities are of interest.

We proceed similarly as in Equations 4-8 of the main text. Leaving the simulated covariates $\boldsymbol{x}^{*}$ at their set values, we fix the value of treatment at z_i^* for all $i = 1, \ldots, N^*$. By plugging treatment A into the Cox regression fit for each simulated unit, we compute the expected marginal survival probability when all subjects are under treatment A:

$$\hat{P}(T_1 > t) = \frac{1}{N^*} \sum_{i=1}^{N^*} \hat{S}_i^{(1)}(t \mid \boldsymbol{x}_i^*) \quad (1)$$

$$= \frac{1}{N^*} \sum_{i=1}^{N^*} \exp[-\hat{H}_0(t))]^{\exp(\hat{\beta}_0 + \boldsymbol{x}_i^* \hat{\boldsymbol{\beta}}_{\mathbf{1}} + \hat{\beta}_z + \boldsymbol{x}_i^{*(\boldsymbol{EM})} \hat{\boldsymbol{\beta}}_{\mathbf{2}})}. \quad (2)$$

Above, t denotes a particular time point and T_1 denotes a potential event time under treatment A, such that $\hat{P}(T_1 > t)$ is the mean treatment-specific probability of surviving beyond t. In Equation 1, $\hat{S}_i^{(1)}(t \mid \boldsymbol{x}_i^*)$ denotes an estimate of the survival probability under treatment A at time t for simulated subject i with covariates $\boldsymbol{x}_i^*$. Equation 2 follows from expressing the survival function in terms of $\hat{H}_0(t)$, an estimate of the baseline cumulative hazard function at time t, exponentiated and raised to the power of the exponentiated linear predictor term. Estimates of the baseline cumulative hazard are easily obtained from Cox regressions fitted with the standard survival analysis software packages.

Similarly, the expected marginal survival probability when all simulated subjects are

under treatment C is given by:

$$\hat{P}(T_0 > t) = \frac{1}{N^*} \sum_{i=1}^{N^*} \hat{S}_i^{(0)}(t \mid \boldsymbol{x}_i^*) \tag{3}$$

$$= \frac{1}{N^*} \sum_{i=1}^{N^*} \exp[-\hat{H}_0(t)]^{\exp(\hat{\beta}_0 + \boldsymbol{x}_i^* \hat{\boldsymbol{\beta}}_1)}, \tag{4}$$

where T_0 denotes a potential event time under treatment C, and $\hat{S}_i^{(0)}(t \mid \boldsymbol{x}_i^*)$ denotes the estimated survival probability under treatment C at time t for subject i with simulated covariates $\boldsymbol{x}_i^*$. The marginal hazard at time t for treatment $z^* \in \{0, 1\}$ can be expressed as the negative logarithm of the survival probability, $-\ln[\hat{P}(T_{z^*} > t)]$. Therefore, the estimate for the marginal log hazard ratio for A vs. C in the BC population at time t is:

$$\hat{\Delta}_{10,t}^{(2)} = \ln\{-\ln[\hat{P}(T_1 > t)]\} - \ln\{-\ln[\hat{P}(T_0 > t)]\}, \tag{5}$$

where $\hat{P}(T_1 > t)$ and $\hat{P}(T_0 > t)$ are obtained using Equations 1-2 and Equations 3-4, respectively.

The Cox regression assumes that the true marginal log hazard ratio is independent of time due to the proportional hazards assumption. However, as pointed out by Varadhan et al.,[35] the estimate $\hat{\Delta}_{10,t}^{(2)}$ in Equation 5 may vary across different values of t. We have to set t to a specific time point, or alternatively, to estimate the marginal hazard ratio over a set of time points and display the estimates graphically. When selecting a value of t, bear in mind that, in Equation 5, the marginal log hazard ratio estimate is undefined at t for which $\hat{P}(T_{z^*} > t) = 1$ for treatment $z^* \in \{0, 1\}$.[34] A simulation procedure for marginalizing estimates of conditional hazard ratios has recently been proposed by Daniel et al.[36] This approach should avoid these issues by averaging the marginal log hazard ratio over a set time frame, but adapting the methodology to the current setting is beyond the scope of this article.

One can manipulate the expected marginal survival probabilities differently than in Equation 5 to produce estimates of the marginal risk difference (the additive difference in survival probabilities) or the marginal log relative risk at a particular time point.[34] These effect measures are more easily interpreted. However, indirect treatment comparisons with survival outcomes are typically performed in the log hazard ratio scale,[37] and this linear predictor scale is used to define effect modification, which is scale-specific.[2] Therefore, the marginal log hazard ratio is the relative effect measure of interest.

Example `R` code implementing the method using maximum-likelihood estimation on a simulated example is provided in Appendix D of the Supplementary Material. Bayesian parametric G-computation would follow a similar approach, and would involve drawing the marginal survival probabilities under each treatment from their posterior predictive distribution. Implementing Bayesian parametric G-computation in the Cox regression scenario is a research priority.

Supplementary Appendix D: Example code

Example R code implementing MAIC, the conventional STC, maximum-likelihood parametric G-computation and Bayesian parametric G-computation on a simulated dataset is provided below. The code and data are available at https://github.com/remiroazocar/Gcomp_indirect_comparisons_simstudy in the Example subdirectory. Full code for implementing the simulation study is available in the online repository.

The simulation study uses binary outcomes and a logistic regression outcome model. Nevertheless, all methods are general-purpose frameworks that, under a generalized linear modeling formulation, can be easily adapted to different outcome models, outcome types, and scalar measures of treatment effect. The code below can be altered by changing the link function in the outcome model. For instance: (1) for a normal linear regression, by setting family=gaussian in the arguments to the glm (or stanglm) function, such that the link is the identity function (for the weighted outcome model, in the case of MAIC); (2) for a Gamma regression, set family=Gamma, and, for parametric G-computation, transform the predicted marginal outcome means to the linear predictor scale using the "negative inverse" link ($g(\mu) = -\mu^{-1}$, for outcome mean μ); (3) for a Poisson regression, set family=poisson, and, for parametric G-computation, transform the marginal outcome means to the linear predictor scale using the log link ($g(\mu) = \ln(\mu)$); and (4) for an inverse Gaussian regression, set family=inverse.gaussian, and, for parametric G-computation, transform the marginal outcome means to the linear predictor scale using the "inverse squared" link ($g(\mu) = \mu^{-2}$).

MAIC

```
library("boot") # for non-parametric bootstrap

AC.IPD <- read.csv("Example/AC_IPD.csv") # load AC patient-level data
BC.ALD <- read.csv("Example/BC_ALD.csv") # load BC aggregate-level data

set.seed(555) # set seed for reproducibility

# objective function to be minimized for standard method of moments
Q <- function(alpha, X.EM) {
  return(sum(exp(X.EM %*% alpha)))
}

# function to be bootstrapped
maic.boot <- function(data, indices) {
  dat <- data[indices,] # AC bootstrap sample
  N <- nrow(dat) # number of subjects in sample
  x.EM <- dat[,c("X1","X2")] # AC effect modifiers
  # BC effect modifier means, assumed fixed
  theta <- BC.ALD[c("mean.X1", "mean.X2")]
  K.EM <- ncol(x.EM) # number of effect modifiers
  # center the AC effect modifiers on the BC means
  x.EM$X1 <- x.EM$X1 - theta$mean.X1
  x.EM$X2 <- x.EM$X2 - theta$mean.X2
  # MAIC weight estimation using method of moments
```

```
  alpha <- rep(1,K.EM) # arbitrary starting point for the optimizer
  # objective function minimized using BFGS
  Q.min <- optim(fn=Q, X.EM=as.matrix(x.EM), par=alpha, method="BFGS")
  # finite solution is the logistic regression parameters
  hat.alpha <- Q.min$par
  log.hat.w <- rep(0, N)
  for (k in 1:K.EM) {
    log.hat.w <- log.hat.w + hat.alpha[k]*x.EM[,k]
  }
  hat.w <- exp(log.hat.w) # estimated weights
  # fit weighted logistic regression model using glm
  outcome.fit <- glm(y~trt, family="quasibinomial", weights=hat.w,
                     data=dat)
  # fitted treatment coefficient is marginal effect for A vs. C
  hat.Delta.AC <- coef(outcome.fit)["trt"]
  return(hat.Delta.AC)
}

# non-parametric bootstrap with 1000 resamples
boot.object <- boot::boot(data=AC.IPD, statistic=maic.boot, R=1000)
# bootstrap mean of marginal A vs. C treatment effect estimate
hat.Delta.AC <- mean(boot.object$t)
# bootstrap variance of A vs. C treatment effect estimate
hat.var.Delta.AC <- var(boot.object$t)
# B vs. C marginal treatment effect from reported event counts
hat.Delta.BC <- with(BC.ALD, log(y.B.sum*(N.C-y.C.sum)/
                                   (y.C.sum*(N.B-y.B.sum))))
# B vs. C marginal effect variance using the delta method
hat.var.Delta.BC <- with(BC.ALD, 1/y.C.sum+1/(N.C-y.C.sum)+
                             1/y.B.sum+1/(N.B-y.B.sum))
hat.Delta.AB <- hat.Delta.AC - hat.Delta.BC # A vs. B
hat.var.Delta.AB <- hat.var.Delta.AC + hat.var.Delta.BC
# construct Wald-type normal distribution-based confidence interval
uci.Delta.AB <- hat.Delta.AB + qnorm(0.975)*sqrt(hat.var.Delta.AB)
lci.Delta.AB <- hat.Delta.AB + qnorm(0.025)*sqrt(hat.var.Delta.AB)
```

Conventional STC

```
AC.IPD <- read.csv("Example/AC_IPD.csv") # load AC patient-level data
BC.ALD <- read.csv("Example/BC_ALD.csv") # load BC aggregate-level data

# fit regression model of outcome on treatment and covariates
# IPD effect modifiers centered at the mean BC values
# purely prognostic variables are included but not centered
outcome.model <- glm(y~X3+X4+trt*I(X1-BC.ALD$mean.X1)+
                       trt*I(X2-BC.ALD$mean.X2),
                     data=AC.IPD, family=binomial)
# fitted treatment coefficient is relative A vs. C conditional effect
hat.Delta.AC <- coef(outcome.model)["trt"]
# estimated variance for A vs. C from model fit
hat.var.Delta.AC <- vcov(outcome.model)["trt", "trt"]
# B vs. C marginal treatment effect estimated from reported event counts
hat.Delta.BC <- with(BC.ALD, log(y.B.sum*(N.C-y.C.sum)/
                                   (y.C.sum*(N.B-y.B.sum))))
```

```
# B vs. C marginal treatment effect variance using the delta method
hat.var.Delta.BC <- with(BC.ALD, 1/y.C.sum+1/(N.C-y.C.sum)+
                           1/y.B.sum+1/(N.B-y.B.sum))
hat.Delta.AB <- hat.Delta.AC - hat.Delta.BC # A vs. B
hat.var.Delta.AB <- hat.var.Delta.AC + hat.var.Delta.BC
# construct Wald-type normal distribution-based confidence interval
uci.Delta.AB <- hat.Delta.AB + qnorm(0.975)*sqrt(hat.var.Delta.AB)
lci.Delta.AB <- hat.Delta.AB + qnorm(0.025)*sqrt(hat.var.Delta.AB)
```

Maximum-likelihood parametric G-computation

```
library("copula") # for simulating BC covariates from Gaussian copula
library("boot") # for non-parametric bootstrap

AC.IPD <- read.csv("Example/AC_IPD.csv") # load AC patient-level data
BC.ALD <- read.csv("Example/BC_ALD.csv") # load BC aggregate-level data

set.seed(555) # set seed for reproducibility

# matrix of pairwise correlations between IPD covariates
rho <- cor(AC.IPD[,c("X1","X2","X3","X4")])
#  covariate simulation for BC trial using copula package
cop <- normalCopula(param=c(rho[1,2],rho[1,3],rho[1,4],rho[2,3],
                            rho[2,4],rho[3,4]),
                    dim=4, dispstr="un") # AC IPD pairwise correlations
# sample covariates from approximate joint distribution using copula
mvd <- mvdc(copula=cop, margins=c("norm", "norm", # Gaussian marginals
                                  "norm", "norm"),
            # BC covariate means and standard deviations
            paramMargins=list(list(mean=BC.ALD$mean.X1, sd=BC.ALD$sd.X1),
                              list(mean=BC.ALD$mean.X2, sd=BC.ALD$sd.X2),
                              list(mean=BC.ALD$mean.X3, sd=BC.ALD$sd.X3),
                              list(mean=BC.ALD$mean.X4, sd=BC.ALD$sd.X4)))
# simulated BC pseudo-population of size 1000
x_star <- as.data.frame(rMvdc(1000, mvd))
colnames(x_star) <- c("X1", "X2", "X3", "X4")
# this function will be bootstrapped
gcomp.ml <- function(data, indices) {
  dat = data[indices,]
  # outcome logistic regression fitted to IPD using maximum likelihood
  outcome.model <- glm(y~X3+X4+trt*X1+trt*X2, data=dat, family=binomial)
  # counterfactual datasets
  data.trtA <- data.trtC <- x_star
  # intervene on treatment while keeping set covariates fixed
  data.trtA$trt <- 1 # dataset where everyone receives treatment A
  data.trtC$trt <- 0 # dataset where all observations receive C
  # predict counterfactual event probs, conditional on treatment/covariates
  hat.mu.A.i <- predict(outcome.model, type="response", newdata=data.trtA)
  hat.mu.C.i <- predict(outcome.model, type="response", newdata=data.trtC)
  hat.mu.A <- mean(hat.mu.A.i) # (marginal) mean probability prediction
     under A
  hat.mu.C <- mean(hat.mu.C.i) # (marginal) mean probability prediction
     under C
  # marginal A vs. C log-odds ratio (mean difference in expected log-odds)
```

```
  # estimated by transforming from probability to linear predictor scale
  hat.Delta.AC <- log(hat.mu.A/(1-hat.mu.A)) - log(hat.mu.C/(1-hat.mu.C))
  # hat.Delta.AC <- qlogis(hat.mu.A) - qlogis(hat.mu.C)
  return(hat.Delta.AC)
}
# non-parametric bootstrap with 1000 resamples
boot.object <- boot::boot(data=AC.IPD, statistic=gcomp.ml, R=1000)
# bootstrap mean of marginal A vs. C treatment effect estimate
hat.Delta.AC <- mean(boot.object$t)
# bootstrap variance of A vs. C treatment effect estimate
hat.var.Delta.AC <- var(boot.object$t)
# marginal log-odds ratio for B vs. C from reported event counts
hat.Delta.BC <- with(BC.ALD,log(y.B.sum*(N.C-y.C.sum)/
                                  (y.C.sum*(N.B-y.B.sum))))
# variance of B vs. C using delta method
hat.var.Delta.BC <- with(BC.ALD,1/y.C.sum+1/(N.C-y.C.sum)+
                           1/y.B.sum+1/(N.B-y.B.sum))
# marginal treatment effect for A vs. B
hat.Delta.AB <- hat.Delta.AC - hat.Delta.BC
# variance for A vs. B
hat.var.Delta.AB <- hat.var.Delta.AC + hat.var.Delta.BC
# construct Wald-type normal distribution-based confidence interval
uci.Delta.AB <- hat.Delta.AB + qnorm(0.975)*sqrt(hat.var.Delta.AB)
lci.Delta.AB <- hat.Delta.AB + qnorm(0.025)*sqrt(hat.var.Delta.AB)
```

Bayesian parametric G-computation

```
library("copula") # for simulating BC covariates from Gaussian copula
# for outcome regression and drawing outcomes from posterior predictive
   dist.
library("rstanarm")

AC.IPD <- read.csv("Example/AC_IPD.csv") # load AC patient-level data
BC.ALD <- read.csv("Example/BC_ALD.csv") # load BC aggregate-level data

set.seed(555) # set seed for reproducibility

# matrix of pairwise correlations between IPD covariates
rho <- cor(AC.IPD[,c("X1","X2","X3","X4")])
#  covariate simulation for BC trial using copula package
cop <- normalCopula(param=c(rho[1,2],rho[1,3],rho[1,4],rho[2,3],
                            rho[2,4],rho[3,4]),
                    dim=4, dispstr="un") # AC IPD pairwise correlations
# sample covariates from approximate joint distribution using copula
mvd <- mvdc(copula=cop, margins=c("norm", "norm", # Gaussian marginals
                                  "norm", "norm"),
            # BC covariate means and standard deviations
            paramMargins=list(list(mean=BC.ALD$mean.X1, sd=BC.ALD$sd.X1),
                              list(mean=BC.ALD$mean.X2, sd=BC.ALD$sd.X2),
                              list(mean=BC.ALD$mean.X3, sd=BC.ALD$sd.X3),
                              list(mean=BC.ALD$mean.X4, sd=BC.ALD$sd.X4)))
# simulated BC pseudo-population of size 1000
x_star <- as.data.frame(rMvdc(1000, mvd))
colnames(x_star) <- c("X1", "X2", "X3", "X4")
```

```
# outcome logistic regression fitted to IPD using MCMC (Stan)
outcome.model <- stan_glm(y~X3+X4+trt*X1+trt*X2, data=AC.IPD,
                          family=binomial, algorithm="sampling",
                          iter=4000, warmup=2000, chains=2)
# counterfactual datasets
data.trtA <- data.trtC <- x_star
# intervene on treatment while keeping set covariates fixed
data.trtA$trt <- 1 # dataset where everyone receives treatment A
data.trtC$trt <- 0 # dataset where all observations receive C
# draw binary responses from posterior predictive distribution
# matrix of posterior predictive draws under A
y.star.A <- posterior_predict(outcome.model, newdata=data.trtA)
# matrix of posterior predictive draws under C
y.star.C <- posterior_predict(outcome.model, newdata=data.trtC)
# compute marginal log-odds ratio for A vs. C for each MCMC sample
# by transforming from probability to linear predictor scale
hat.delta.AC <- qlogis(rowMeans(y.star.A)) - qlogis(rowMeans(y.star.C))
hat.Delta.AC <- mean(hat.delta.AC) # average over samples
hat.var.Delta.AC <- var(hat.delta.AC) # sample variance
# B vs. C from reported aggregate event counts in contingency table
hat.Delta.BC <- with(BC.ALD, log(y.B.sum*(N.C-y.C.sum)/
                                   (y.C.sum*(N.B-y.B.sum))))
# B vs. C variance using the delta method
hat.var.Delta.BC <- with(BC.ALD, 1/y.C.sum+1/(N.C-y.C.sum)+
                           1/y.B.sum+1/(N.B-y.B.sum))
# marginal treatment effect for A vs. B
hat.Delta.AB <- hat.Delta.AC - hat.Delta.BC
# A vs. B variance
hat.var.Delta.AB <- hat.var.Delta.AC + hat.var.Delta.BC
# construct Wald-type normal distribution-based confidence interval
uci.Delta.AB <- hat.Delta.AB + qnorm(0.975)*sqrt(hat.var.Delta.AB)
lci.Delta.AB <- hat.Delta.AB + qnorm(0.025)*sqrt(hat.var.Delta.AB)
```

Cox regression: Maximum-likelihood parametric G-computation

Below, we provide example R code implementing parametric G-computation with survival outcomes and Cox regression as the outcome model. We use maximum-likelihood estimation to fit the multivariable Cox regression, then predicting the outcomes on the BC population. Variance estimation for the marginal A vs. C treatment effect is performed by resampling via the ordinary non-parametric bootstrap.

```
library("survival") # to fit Cox proportional hazards regression
library("copula") # for simulating BC covariates from Gaussian copula
library("boot") # for non-parametric bootstrap

AC.IPD <- read.csv("Example/Survival/AC_IPD_survival.csv") # load AC
    patient-level data
BC.ALD <- read.csv("Example/Survival/BC_ALD_survival.csv") # load BC
    aggregate-level data

set.seed(555) # set seed for reproducibility

# matrix of pairwise correlations between IPD covariates
```

```
rho <- cor(AC.IPD[,c("X1","X2","X3","X4")])
#  covariate simulation for BC trial using copula package
cop <- normalCopula(param=c(rho[1,2],rho[1,3],rho[1,4],rho[2,3],
                            rho[2,4],rho[3,4]),
                    dim=4, dispstr="un") # AC IPD pairwise correlations
# sample covariates from approximate joint distribution using copula
mvd <- mvdc(copula=cop, margins=c("norm", "norm", # Gaussian marginals
                                  "norm", "norm"),
            # BC covariate means and standard deviations
            paramMargins=list(list(mean=BC.ALD$mean.X1, sd=BC.ALD$sd.X1),
                              list(mean=BC.ALD$mean.X2, sd=BC.ALD$sd.X2),
                              list(mean=BC.ALD$mean.X3, sd=BC.ALD$sd.X3),
                              list(mean=BC.ALD$mean.X4, sd=BC.ALD$sd.X4)))
# simulated BC pseudo-population of size 1000
x_star <- as.data.frame(rMvdc(1000, mvd))
colnames(x_star) <- c("X1", "X2", "X3", "X4")

# function to be resampled by non-parametric bootstrap
gcomp.ml <- function(data, indices) {
  dat = data[indices,]
  # outcome Cox regression model fitted to IPD using maximum likelihood
  outcome.model <- coxph(Surv(time, status)~trt*X1+trt*X2+X3+X4, data=dat)
  # event time selected for unit 50 (random selection)
  unit.time <- 50
  # estimated cumulative baseline hazard
  hat.H0 <- basehaz(outcome.model)[unit.time,1]
  # counterfactual datasets (two hypothetical worlds)
  data.trtA <- data.trtC <- x_star
  # intervene on treatment while keeping set covariates fixed
  data.trtA$trt <- 1 # dataset where everyone receives treatment A
  data.trtC$trt <- 0 # dataset where all observations receive C
  # linear predictor where everyone receives treatment A
  LP.A <- with(outcome.model, x_star$X1*(coefficients["X1"] + coefficients
      ["trt:X1"]) +
                 x_star$X2*(coefficients["X2"] + coefficients["trt:X2"]) +
                 x_star$X3*coefficients["X3"] + x_star$X4*coefficients["X4
                     "] +
                 coefficients["trt"])
  # linear predictor where all observations receive treatment C
  LP.C <- with(outcome.model, x_star$X1*coefficients["X1"] + x_star$X2*
      coefficients["X2"] +
                 x_star$X3*coefficients["X3"] + x_star$X4*coefficients["X4
                     "])
  # predict individual survival probabilities, conditional on treatment/
      covariates
  hat.S.A.i <- exp(-hat.H0)^exp(LP.A)
  hat.S.C.i <- exp(-hat.H0)^exp(LP.C)
  # mean survival probability prediction under each treatment
  hat.P.A <- mean(hat.S.A.i)
  hat.P.C <- mean(hat.S.C.i)
  # estimate marginal A vs. B log hazard ratio (mean difference in expected
       log hazard)
  # by transforming from survival probability to linear predictor scale
  hat.Delta.AC <- log(-log(hat.P.A)) - log(-log(hat.P.C))
  return(hat.Delta.AC)
```

```
}
# non-parametric bootstrap with 1000 resamples (ignore warnings)
boot.object <- boot::boot(data=AC.IPD, statistic=gcomp.ml, R=1000)
# bootstrap mean of marginal A vs. C treatment effect estimate
hat.Delta.AC <- mean(boot.object$t)
# bootstrap variance of A vs. C treatment effect estimate
hat.var.Delta.AC <- var(boot.object$t)
# marginal log hazard ratio for B vs. C reported in BC article
hat.Delta.BC <- BC.ALD$logHR_B
# variance of B vs. C in aggregate outcomes in published article
hat.var.Delta.BC <- BC.ALD$var_logHR_B
# marginal treatment effect for A vs. B
hat.Delta.AB <- hat.Delta.AC - hat.Delta.BC
# variance for A vs. B
hat.var.Delta.AB <- hat.var.Delta.AC + hat.var.Delta.BC
# construct Wald-type normal distribution-based confidence interval
uci.Delta.AB <- hat.Delta.AB + qnorm(0.975)*sqrt(hat.var.Delta.AB)
lci.Delta.AB <- hat.Delta.AB + qnorm(0.025)*sqrt(hat.var.Delta.AB)
```

References

[1] James E Signorovitch, Eric Q Wu, P Yu Andrew, Charles M Gerrits, Evan Kantor, Yanjun Bao, Shiraz R Gupta, and Parvez M Mulani. Comparative effectiveness without head-to-head trials. *Pharmacoeconomics*, 28(10):935–945, 2010.

[2] David Phillippo, Tony Ades, Sofia Dias, Stephen Palmer, Keith R Abrams, and Nicky Welton. Nice dsu technical support document 18: methods for population-adjusted indirect comparisons in submissions to nice. 2016.

[3] Antonio Remiro-Azócar, Anna Heath, and Gianluca Baio. Methods for population adjustment with limited access to individual patient data: A review and simulation study. *arXiv preprint arXiv:2004.14800*, 2020.

[4] David M Phillippo, Sofia Dias, AE Ades, and Nicky J Welton. Equivalence of entropy balancing and the method of moments for matching-adjusted indirect comparison. *Research Synthesis Methods*, 2020.

[5] Bradley Efron and Robert J Tibshirani. *An introduction to the bootstrap.* CRC press, 1994.

[6] Halbert White et al. A heteroskedasticity-consistent covariance matrix estimator and a direct test for heteroskedasticity. *econometrica*, 48(4):817–838, 1980.

[7] Göran Kauermann and Raymond J Carroll. A note on the efficiency of sandwich covariance matrix estimation. *Journal of the American Statistical Association*, 96(456):1387–1396, 2001.

[8] Helmut Petto, Zbigniew Kadziola, Alan Brnabic, Daniel Saure, and Mark Belger. Alternative weighting approaches for anchored matching-adjusted indirect comparisons via a common comparator. *Value in Health*, 22(1):85–91, 2019.

[9] M Belger, A Brnabic, Z Kadziola, H Petto, and D Faries. Inclusion of multiple studies in matching adjusted indirect comparisons (maic). *Value in Health*, 18(3):A33, 2015.

[10] Frank Windmeijer. A finite sample correction for the variance of linear efficient two-step gmm estimators. *Journal of econometrics*, 126(1):25–51, 2005.

[11] Anthony James Hatswell, Nick Freemantle, and Gianluca Baio. The effects of model misspecification in unanchored matching-adjusted indirect comparison (maic): Results of a simulation study. *Value in Health*, 2020.

[12] David M Phillippo, Sofia Dias, AE Ades, and Nicky J Welton. Assessing the performance of population adjustment methods for anchored indirect comparisons: A simulation study. *Statistics in Medicine*, 2020.

[13] David M Phillippo, Sofia Dias, AE Ades, and Nicky J Welton. Target estimands for efficient decision making: Response to comments on "assessing the performance of population adjustment methods for anchored indirect comparisons: A simulation study". *Statistics in Medicine*, 2021.

[14] Rajeev H Dehejia and Sadek Wahba. Causal effects in nonexperimental studies: Reevaluating the evaluation of training programs. *Journal of the American statistical Association*, 94(448):1053–1062, 1999.

[15] Ingeborg Waernbaum. Propensity score model specification for estimation of average treatment effects. *Journal of Statistical Planning and Inference*, 140(7):1948–1956, 2010.

[16] Ben Goodrich, Jonah Gabry, Imad Ali, and Sam Brilleman. rstanarm: Bayesian applied regression modeling via stan. *R package version*, 2(4):1758, 2018.

[17] Stan Developent Team et al. Rstan: the r interface to stan. *R package version*, 2(1):522, 2016.

[18] Bob Carpenter, Andrew Gelman, Matthew D Hoffman, Daniel Lee, Ben Goodrich, Michael Betancourt, Marcus Brubaker, Jiqiang Guo, Peter Li, and Allen Riddell. Stan: A probabilistic programming language. *Journal of statistical software*, 76(1), 2017.

[19] Andrew Gelman, Aleks Jakulin, Maria Grazia Pittau, Yu-Sung Su, et al. A weakly informative default prior distribution for logistic and other regression models. *The annals of applied statistics*, 2(4):1360–1383, 2008.

[20] Andrew Gelman, John B Carlin, Hal S Stern, David B Dunson, Aki Vehtari, and Donald B Rubin. *Bayesian data analysis*. CRC press, 2013.

[21] J Martin Bland and Douglas G Altman. The odds ratio. *Bmj*, 320(7247):1468, 2000.

[22] Erin Hartman, Richard Grieve, Roland Ramsahai, and Jasjeet S Sekhon. From sample average treatment effect to population average treatment effect on the treated: combining experimental with observational studies to estimate population treatment effects. *Journal of the Royal Statistical Society: Series A (Statistics in Society)*, 178(3):757–778, 2015.

[23] Antonio Remiro-Azócar, Anna Heath, and Gianluca Baio. Principled selection of effect modifiers: Comments on'matching-adjusted indirect comparisons: Application to time-to-event data'. *arXiv preprint arXiv:2012.05127*, 2020.

[24] Trang Quynh Nguyen, Cyrus Ebnesajjad, Stephen R Cole, and Elizabeth A Stuart. Sensitivity analysis for an unobserved moderator in rct-to-target-population generalization of treatment effects. *The Annals of Applied Statistics*, pages 225–247, 2017.

[25] Issa J Dahabreh. *Extending Causal Inferences From a Randomized Trial to Another Population*. PhD thesis, 2019.

[26] Issa J Dahabreh, Sarah E Robertson, Jon A Steingrimsson, Elizabeth A Stuart, and Miguel A Hernan. Extending inferences from a randomized trial to a new target population. *Statistics in medicine*, 39(14):1999–2014, 2020.

[27] Jared K Lunceford and Marie Davidian. Stratification and weighting via the propensity score in estimation of causal treatment effects: a comparative study. *Statistics in medicine*, 23(19):2937–2960, 2004.

[28] Alexander P Keil, Julie L Daniels, and Irva Hertz-Picciotto. Autism spectrum disorder, flea and tick medication, and adjustments for exposure misclassification: the charge (childhood autism risks from genetics and environment) case–control study. *Environmental Health*, 13(1):1–10, 2014.

[29] David Madigan and Adrian E Raftery. Model selection and accounting for model uncertainty in graphical models using occam's window. *Journal of the American Statistical Association*, 89(428):1535–1546, 1994.

[30] Dennis O Dixon and Richard Simon. Bayesian subset analysis. *Biometrics*, pages 871–881, 1991.

[31] David J Spiegelhalter, Laurence S Freedman, and Mahesh KB Parmar. Bayesian approaches to randomized trials. *Journal of the Royal Statistical Society: Series A (Statistics in Society)*, 157(3):357–387, 1994.

[32] Richard Simon and Laurence S Freedman. Bayesian design and analysis of two x two factorial clinical trials. *Biometrics*, pages 456–464, 1997.

[33] David M Phillippo, Sofia Dias, Ahmed Elsada, AE Ades, and Nicky J Welton. Population adjustment methods for indirect comparisons: A review of national institute for health and care excellence technology appraisals. *International journal of technology assessment in health care*, pages 1–8, 2019.

[34] Ori M Stitelman, C William Wester, Victor De Gruttola, and Mark J van der Laan. Targeted maximum likelihood estimation of effect modification parameters in survival analysis. *The international journal of biostatistics*, 7(1), 2011.

[35] Ravi Varadhan, Nicholas C Henderson, and Carlos O Weiss. Cross-design synthesis for extending the applicability of trial evidence when treatment effect is heterogeneous: Part i. methodology. *Communications in Statistics: Case Studies, Data Analysis and Applications*, 2(3-4):112–126, 2016.

[36] Rhian Daniel, Jingjing Zhang, and Daniel Farewell. Making apples from oranges: Comparing noncollapsible effect estimators and their standard errors after adjustment for different covariate sets. *Biometrical Journal*, 2020.

[37] Sofia Dias, Alex J Sutton, AE Ades, and Nicky J Welton. Evidence synthesis for decision making 2: a generalized linear modeling framework for pairwise and network meta-analysis of randomized controlled trials. *Medical Decision Making*, 33(5):607–617, 2013.
